# Correlation of histopathology and multi-modal magnetic resonance imaging in childhood osteosarcoma: Predicting tumor response to chemotherapy

**DOI:** 10.1371/journal.pone.0259564

**Published:** 2022-02-14

**Authors:** Ka Yaw Teo, Ovidiu Daescu, Kevin Cederberg, Anita Sengupta, Patrick J. Leavey

**Affiliations:** 1 Department of Computer Science, University of Texas at Dallas, Richardson, Texas, United States of America; 2 Department of Radiology, University of Texas Southwestern Medical Center, Dallas, Texas, United States of America; 3 Department of Pathology, University of Texas Southwestern Medical Center, Dallas, Texas, United States of America; 4 Department of Pediatrics, University of Texas Southwestern Medical Center, Dallas, Texas, United States of America; University College London, UNITED KINGDOM

## Abstract

**Background:**

Osteosarcoma, which is the most common malignant pediatric bone cancer, remains dependent on an imprecise systemic treatment largely unchanged in 30 years. In this study, we correlated histopathology with magnetic resonance imaging (MRI), used the correlation to extract MRI-specific features representative of tumor necrosis, and subsequently developed a novel classification model for predicting tumor response to neoadjuvant chemotherapy in pediatric patients with osteosarcoma using multi-modal MRI. The model could ultimately serve as a testable biomarker for a high-risk malignancy without successful precision treatments.

**Methods:**

Patients with newly diagnosed high-grade appendicular osteosarcoma were enrolled in a single-center observational study, wherein patients underwent pre-surgical evaluation using both conventional MRI (post-contrast T1-weighted with fat saturation, pre-contrast T1-weighted, and short inversion-time inversion recovery (STIR)) and advanced MRI (diffusion weighted (DW) and dynamic contrast enhanced (DCE)). A classification model was established based on a direct correlation between histopathology and MRI, which was achieved through histologic-MR image co-registration and subsequent extraction of MR image features for identifying histologic tumor necrosis. By operating on the MR image features, tumor necrosis was estimated from different combinations of MR images using a multi-feature fuzzy clustering technique together with a weighted majority ruling. Tumor necrosis calculated from MR images, for either an MRI plane of interest or whole tumor volume, was compared to pathologist-estimated necrosis and necrosis quantified from digitized histologic section images using a previously described deep learning classification method.

**Results:**

15 patients were enrolled, of whom two withdrew, one became ineligible, and two were subjected to inadequate pre-surgical imaging. MRI sequences of *n* = 10 patients were subsequently used for classification model development. Different MR image features, depending on the modality of MRI, were shown to be significant in distinguishing necrosis from viable tumor. The scales at which MR image features optimally signified tumor necrosis were different as well depending on the MR image type. Conventional MRI was shown capable of differentiating necrosis from viable tumor with an accuracy averaging above 90%. Conventional MRI was equally effective as DWI in distinguishing necrotic from viable tumor regions. The accuracy of tumor necrosis prediction by conventional MRI improved to above 95% when DCE-MRI was added into consideration. Volume-based tumor necrosis estimations tended to be lower than those evaluated on an MRI plane of interest.

**Conclusions:**

The study has shown a proof-of-principle model for interpreting chemotherapeutic response using multi-modal MRI for patients with high-grade osteosarcoma. The model will continue to be evaluated as MR image features indicative of tumor response are now computable for the disease prior to surgery.

## Introduction

Osteosarcoma is the most common type of malignant bone cancer prevalent in children and adolescents. Comprising 60% of all bone sarcomas [1–3], patients with high-grade osteosarcoma typically receive a treatment schedule of pre-operative chemotherapy followed by surgical resection of the primary tumor followed by further post-operative chemotherapy. Treatment response evaluated at time of surgery predicts outcome for patients with high-grade osteosarcoma [4, 5], and efforts continue to deliver response-adapted therapy to patients with this high-grade malignancy. This manuscript will refer to the histological high-grade subtype of osteosarcoma, which will hereafter be simply referred to as osteosarcoma.

For many malignancies, an effective course of chemotherapy is traditionally associated with a shrinkage of tumor, which can be serially monitored through axial magnetic resonance imaging (MRI) and computerized tomography (CT), and can be measured by standard criteria such as RECIST 1.1 [6]. However, size of tumor is often not a reliable indicator of the efficacy of chemotherapy for osteosarcoma because the tumor typically fails to reduce in size due to its mineralized matrix not being affected by cytotoxic agents [7]. To evaluate the effectiveness of chemotherapy, surgically resected tumor is analyzed histologically for its degree of necrosis after pre-operative chemotherapy is completed. Chemotherapy is considered successful if the histological grading of the tumor is greater than 90% necrosis. Serial and early determination of treatment response has been unachievable to date for patients with osteosarcoma.

Axial imaging with conventional multi-planar MRI sequences–such as non-contrast T1-weighted, fat-suppressed post-contrast T1-weighted, and short inversion-time inversion recovery (STIR)–remains of importance for the care of patients with osteosarcoma due to its ability to produce images with high tissue contrast and anatomical details. Such image detail is relevant to surgical planning and useful in indicating tumor growth, if it occurs, despite pre-operative chemotherapy. Features such as signal intensity and enhancement patterns after injection of contrast agents [8, 9] are also relevant to surgical planning, and while the evolution of such patterns can be associated with cytotoxic effect, they remain an unreliable predictor of tumor response and patient outcome when evaluated by the naked eye [10].

Advanced MR quantitative parameters have shown promise as potential biomarkers of treatment effect for osseous tumors. Diffusion-weighted imaging (DWI) and dynamic contrast-enhanced MRI (DCE-MRI) are conceptually attractive MRI biomarker techniques, given their relative widespread availability, non-invasiveness, and repeatability. DWI provides information on the Brownian motion of water molecules in tissues, the degree of which is represented by a quantitative measure called apparent diffusion coefficient (ADC) [11, 12]. Areas of restricted diffusion have a low signal intensity on ADC map. Necrotic areas within tumor are typically associated with an increased local diffusion and thus a high signal intensity on ADC map. Seeing the potential of using ADC as an imaging biomarker for osteosarcoma, DWI has been explored by many investigators [13–26]. Previous results have overall indicated that ADC could be used to identify good responding patients with osteosarcoma, as ADC has been shown to differ markedly between necrosis and viable tumor.

Post-contrast MRI cannot unequivocally distinguish viable from non-viable tumor and inflammation because the static images are acquired several minutes after contrast agent injection, allowing time for the contrast agent to equilibrate among various enhancing tissues. Viable tumor, revascularized necrotic tissue, reactive hyperemia, and inflammatory tissue could all exhibit post-contrast enhancement [27, 28]. In response to this limitation, early-phase dynamic contrast-enhanced MRI can help to distinguish (non-enhancing) edema from the rapidly enhancing viable tumor. The basic principles of DCE-MRI, as well as its relevance and clinical applications for osteosarcoma, have been previously discussed in articles such as [29–35] (to be elaborated upon later in detail in the Discussion section). As concluded by prior studies [29–35], DCE-MRI sequences, when being properly analyzed quantitatively, could be helpful for identifying osteosarcoma patients responding positively to chemotherapy, as well as for differentiating necrosis from viable tumor.

Clinical trials for patients with newly diagnosed osteosarcoma continue to emphasize the hypothesis that response-adapted therapy is achievable [36]. However, treatment response as estimated by histological examination of tumor necrosis is dependent on a medical procedure largely unchanged in several decades [37] that likely has several limitations. Such limitations include the following: i) it takes 10 weeks to deliver a typical cassette of potentially ineffective pre-operative chemotherapy [36], ii) response examination is typically performed only on a single plane of tumor within a large three-dimensional specimen, iii) specimen processing of osseous matrix involves de-calcification and potential specimen loss, and iv) the procedure for estimation of tumor necrosis is time intensive and subject to significant inter-observer variability [38].

This report describes the development of a novel classification model for predicting tumor response to chemotherapy using multi-modal MRI sequences, and the model is formed on the basis of establishing a direct correlation between histopathology and MRI. The correlation is achieved through histologic-MR image co-registration and subsequent extraction of MR image features representative of tumor necrosis. By operating on those MR image features, tumor necrosis is estimated from MR images using a multi-feature fuzzy clustering technique coupled with a weighted majority ruling. Overall, the current work provides a proof of concept for the feasibility of identifying histologic tumor necrosis in osteosarcoma using statistical classification based on MR image features.

## Materials and methods

### Patients

Patients were recruited from November 2016 to April 2019 into a prospective observation study at Children’s Medical Center Dallas. The study was approved by the institutional review board of the University of Texas Southwestern Medical Center, Dallas, Texas. Patients with newly-diagnosed high-grade respectable osteosarcoma of an extremity were eligible for enrollment in the study. In addition, eligible patients must be of an age between 10 and 30 years, and must be either English- or Spanish-speaking. All enrolled patients or their parents signed informed consent. Newly diagnosed patients were all cared for by a pediatric oncologist, and were identified as potentially eligible through clinical care. A patient was considered ineligible if they i) required sedation for MR imaging, ii) could not undergo MR imaging, iii) had a secondary bone sarcoma or a bone sarcoma in a previously irradiated field, or iv) had a contraindication to contrast agent.

All recruited patients received pre-operative chemotherapy with cisplatin, doxorubicin, and methotrexate based on the ERUAMOS I regimen [36]. Enrolled patients had advanced MRI sequences (DWI and DCE) added to their conventional sequences at the time of pre-operative imaging typically performed after week 10 of chemotherapy. Enrolled patients also had an additional MRI (conventional, DWI, and DCE sequences) performed after the fifth week of chemotherapy.

### Pre-surgical MRI

MRI was performed on a 3-Tesla scanner (Magnetom Skyra, Siemens Healthcare, Erlangen, Germany), and all MR images were acquired in the coronal plane.

#### Conventional MRI

Conventional static MR evaluations included a T1-weighted spin-echo sequence (repetition time (TR) = 600 ms, echo time (TE) = 10 ms, slice thickness = 4 mm, interslice gap = 1 mm, number of excitations (NEX) = 1, flip angle = 150°) and a short inversion-time inversion recovery (STIR) sequence (TR = 3780 ms, TE = 91 ms, inversion time (TI) = 220 ms, slice thickness = 4 mm, interslice gap = 1 mm, NEX = 1, flip angle = 132°). In addition, a post-contrast T1-weighted fast-spin-echo variable-flip-angle (SPACE) fat-saturated sequence (TR = 450 ms, TE = 11 ms, slice thickness = 1 mm, interslice gap = 0 mm, NEX = 1, flip angle = 120°) was acquired after DCE-MRI.

#### Advanced MRI

Readout-segmented echo-planar (RESOLVE) DWI sequences (TR = 5450 ms, TE = 81 ms, slice thickness = 4 mm, interslice gap = 1 mm, NEX = 1 mm, flip angle = 180°) were obtained with *b*-values of 0, 400, and 800 s/mm^2^. DCE-MRI (TR = 5.07 ms, TE = 1.78 ms, slice thickness = 1.5 mm, interslice gap = 0 mm, NEX = 1, flip angle = 15°), by means of a T1-weighted volumetric interpolated breath-hold examination (VIBE) sequence, was performed using the following image acquisition protocol. Immediately after capturing the first phase (i.e., pre-contrast image), a bolus of gadoterate meglumine (Dotarem, Guerbet, Princeton, NJ) was administered intravenously at a constant rate of 2 mL/s by using a power injector. It was then followed by a normal saline flush (20 mL) at a rate of 2 mL/s. After the bolus injection began, DCE images were acquired continuously for three minutes at intervals of 19.5 s.

Other MR imaging parameters such as acquisition matrix size and field of view were chosen based on the size, location, and geometry of tumor, which varied considerably from patient to patient.

### Surgical resection

Surgical care of patients for local-controlled tumor resection were based on surgeon preferences and established standards of surgical care. No research requirements were implemented for surgical specimen collection. Surgical specimens delivered to pathology were prepared based on established standards.

### Histologic examination

#### Pathology

Each surgical specimen was sectioned into approximately 0.5-cm slices in the coronal plane with the aid of a three-dimensional mold prepared from the fifth-week MRI. The primary section was then fixed in 10% buffered formalin, before being decalcified with 10% hydrochloric acid. The section was divided into approximately 2 × 1.5 cm pieces, which were processed into paraffin-embedded blocks in the standard manner. Individual histologic sections were cut from the blocks at 4-μm thickness, mounted on glass slides, and stained with hematoxylin and eosin. The glass slides were manually reviewed by a pathologist, and tumor necrosis was estimated according to standard procedures. Patients with ≥ 90% tumor necrosis were considered as good responders, whereas patients with < 90% tumor necrosis were considered as poor responders. The glass slides were subsequently digitally scanned (Aperio AT Turbo, Leica Biosystems, Vista, CA) for image processing and analysis.

#### Deep learning classification

By utilizing a previously established deep learning classification model [39], digital whole slide images were analyzed for the presence of necrotic and viable tumor tissue. The deep learning classifier yielded, as an output, a tumor viability map for each whole slide image, indicating the areas of necrosis and viable tumor. An image of the entire primary histologic section and its associated tumor viability map were then reconstructed by stitching together the individual whole slide images and their respective tumor viability maps using an image stitching software [40, 41].

### Pre-processing of MR images

The signal intensities of all conventional MR images were standardized using a non-linear transformation procedure [42] to achieve a level of consistency in intensity scale across the MR images. The multi-modal MR images acquired in each patient study were aligned (co-registered) using the DICOM’s patient-based reference coordinate system (RCS). Furthermore, in order to compensate for any image misalignment due to subject motion between scans, a rigid body co-registration was performed in 3D Slicer (open-source software, www.slicer.org).

### Post-processing and evaluation of advanced MR images

#### DWI sequence analysis

Apparent diffusion coefficient (ADC) was calculated from DWI sequences using an inverse relationship between DWI signal and diffusivity, which can be presented by *S*(*b*) = *S*_0_ exp(−*b* · ADC), where *S* is the signal intensity at a prescribed diffusion weighting (*b*-value). Three different *b*-values (i.e., 0, 400, and 800 s/mm^2^) were used to compute the ADC.

#### DCE-MRI sequence analysis

A “subtraction” sequence was at first generated by subtracting the first pre-contrast image from all subsequent images in a DCE-MRI sequence [27]. Two semi-quantitative parametric images–namely, the steepest slope of and the area under the time-intensity curve–were generated as follows. The steepest slope of the time-intensity curve was calculated for each pixel in the subtraction DCE-MRI sequence as (SI_end_−SI_prior_) · 100 / (SI_base_ · *T*), where *T* is the sampling time interval, SI_base_ is the signal intensity of the pixel in the pre-contrast image (before the injection of contrast agent), and SI_prior_ and SI_end_ are the signal intensities of the pixel that differ the most between any two consecutive time points [43]. The area under the time-intensity curve was computed for each pixel over the first 100 seconds of the subtraction DCE-MRI sequence.

In addition, DCE-MRI data was quantitatively analyzed using a two-compartment pharmacokinetic model [44]. The modeling was performed in 3D Slicer using the following parameter settings. An arterial input function (AIF) was defined by a small region in a feeding artery visible in the DCE-MRI sequence. The relaxivity of contrast agent was 0.0039 Lmol^-1^s^-1^ (for Gd-DOTA at 3T). The T1 relaxation time for arterial blood was 1600 ms. The T1 relaxation time for osteosarcoma was set to 1100 ms, a population-based mean value measured previously by Guo et al. [45]. Two pharmacokinetic parametric maps were produced; for each pixel, two quantitative perfusion parameters were computed–i) the transfer constant *K*^trans^ between blood plasma and extravascular extracellular space (EES), and ii) the volume fraction *v*_e_ of EES.

### Tumor segmentation on MR images

In each patient study, tumor was delineated as the volume of interest (VOI) in the post-contrast T1-weighted MRI sequence by a pediatric radiologist (i.e., single-observer approach). The coronal MRI plane that best corresponded to the plane of primary histologic section was identified by a pathologist and a pediatric radiologist. The best-matching MRI plane was considered as the plane of interest (POI). Additionally, in the discussion that follows, the tumor area of interest (AOI) was defined as the area of cross section of the tumor VOI along the POI.

**Fig 1:**
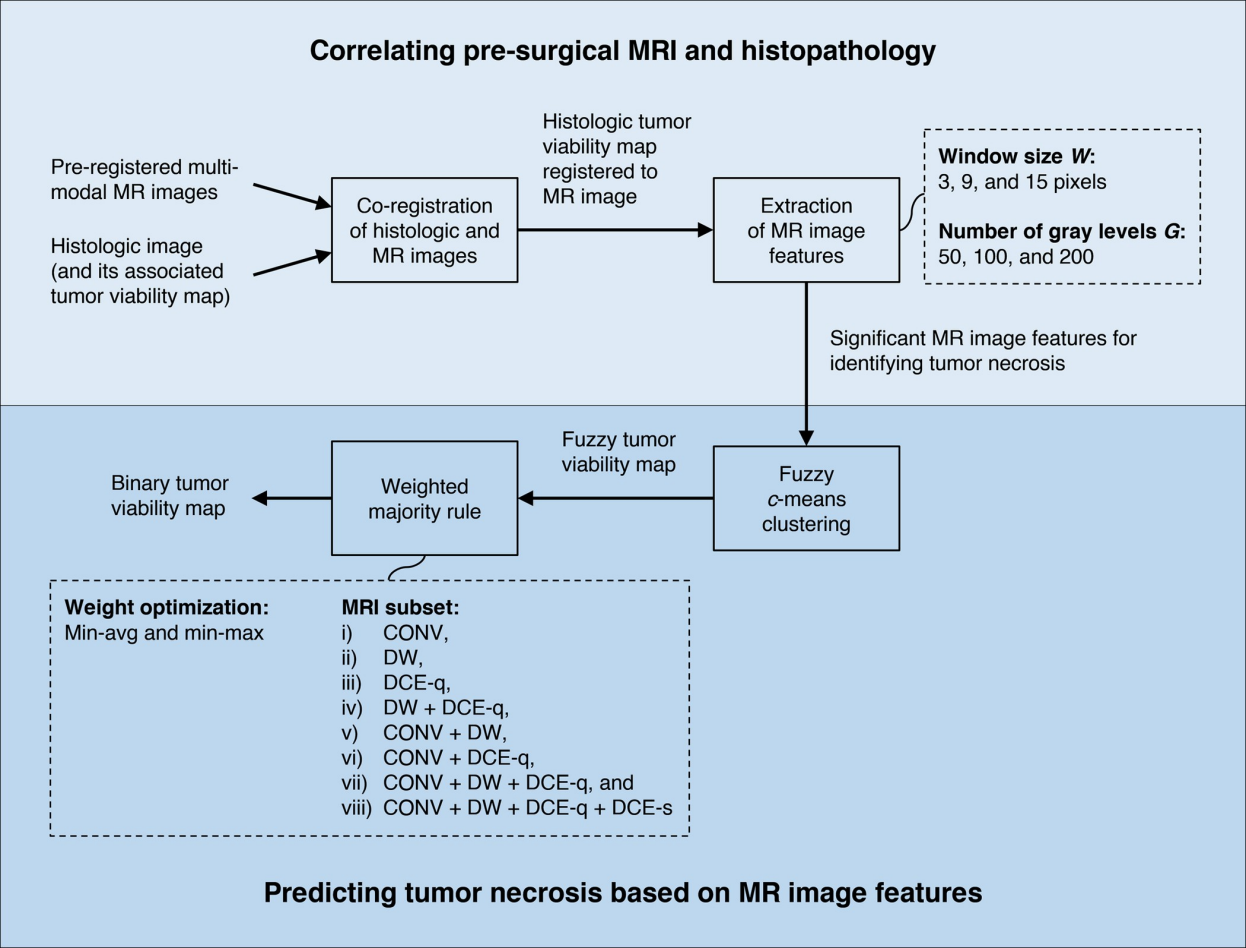
Image processing workflow for correlating MRI with histopathology and predicting tumor necrosis using MRI. Each dashed box specifies the investigated parameters in association with their respective steps in the workflow.

In summary thus far, an image of primary histologic section, its co-registered histologic tumor viability map, and a series of multi-modal MR images, consisting of three conventional MRI sequences and eight advanced MRI derived parametric maps (Table 1), were carefully produced in each patient study to be used in the subsequent process of establishing a classification model for predicting tumor necrosis from MR images. Fig 1 depicts the overall image-processing workflow that follows; it consists of two major stages–i) correlating MRI with histopathology, and ii) predicting tumor necrosis based on MRI. The details of the procedure are given in the following two subsections.

**Table 1:** MR images of different modalities and their derived parametric maps.

No.	Abbreviation	Description
***Conventional (CONV)***		
1	PC	Post-contrast T1-weighted spin-echo fat-saturated
2	T1	T1-weighted spin-echo
3	STIR	Short inversion-time inversion recovery
***Diffusion weighted (DW)***		
4	ADC	Apparent diffusion coefficient
***Dynamic contrast enhanced (DCE)***		
*Subtraction images (DCE-s)*:		
5	DCE-sub-0	Subtraction image at time *t* = 0 s (after contrast arrival)
6	DCE-sub-1	Subtraction image at time *t* = 50 s (after contrast arrival)
7	DCE-sub-2	Subtraction image at time *t* = 100 s (after contrast arrival)
*Quantitative parameter maps (DCE-q)*:		
8	DCE-slope	Steepest slope of the time-intensity curve
9	DCE-AUC	Area under the time-intensity curve
10	DCE-*K*^trans^	Influx volume transfer constant *K*^trans^
11	DCE-*v*_e_	Relative extravascular extracellular space *v*_e_

### Correlation of histopathology and MRI

#### Image co-registration

By employing control point image mapping (MATLAB, MathWorks, Natick, MA), primary histologic section image, together with its corresponding tumor viability map, was registered to each of the pre-aligned multi-modal MR images in the POI. Let AOI_hist_ denote the area of tumor in the histologic image. The main focus herein was to align the tumor AOI_hist_ in the histologic image to the tumor AOI in the MR image. In each patient study, eight to 12 control points, consisting of anatomical landmarks within or in the vicinity of tumor, were defined in the given pair of histologic and MR images by a pathologist and a pediatric radiologist. A geometric mapping was then inferred from the positions of these control points using local weighted mean transformation.

#### Extraction of MR image-based features for identifying tumor necrosis

After co-registering the histologic image to each of the multi-modal MR images in the POI, each pixel in the tumor AOI_hist_ in the MR image can be explicitly categorized as necrotic or viable tumor based on the tumor viability map associated with the co-registered histologic image.

Local image features were then extracted from each of the MR images along the POI as follows. A square window of side length *W* pixels was defined and centered at each pixel in the tumor AOI_hist_. For each such window, four statistical parameters and 19 Haralick texture features were computed (Table 2). Haralick texture features are scalars calculated from a gray level co-occurrence matrix (GLCM) that counts the co-occurrence of neighboring gray levels in the window. Briefly, each texture feature is a function of the elements in the GCLM, representing a specific relation, such as contrast and homogeneity, between neighboring pixels (for detailed definitions of the features, see [46]). The GLCM is a *G* × *G* matrix, where *G* is the number of gray levels. Eight (symmetric) GLCMs were created by considering the closest neighbor in eight directions (i.e., left, right, up, down, and four diagonals). The symmetric GLCMs were summed and normalized to yield a direction invariant GLCM.

**Table 2:** Statistical parameters and Haralick texture features computed from MR images.

No.	Feature
1	Original pixel value
***Statistical features***	
2	Mean
3	Variance
4	Skewness
5	Kurtosis
***Haralick texture features***	
6	Autocorrelation
7	Cluster prominence
8	Cluster shade
9	Contrast
10	Correlation
11	Difference entropy
12	Difference variance
13	Dissimilarity
14	Energy
15	Entropy
16	Homogeneity
17	Information measure of correlation 1
18	Information measure of correlation 2
19	Inverse difference
20	Maximum probability
21	Sum average
22	Sum entropy
23	Sum of squares
24	Sum variance

In order to examine the effects of window size *W* and the number of gray levels *G* on the resulting features and the subsequent performance of the classification model based on those features, the values of *W* and *G* were varied as follows: *W* = 3, 9, or 15, and *G* = 50, 100 or 200. One-way analysis of variance (ANOVA) with unequal sample sizes [47] was used to investigate if any of the features computed were significantly different between necrotic and viable tumor regions when changing the size of the window *W* and the number of gray levels *G*. With respect to its role in differentiating between necrotic and viable tumor regions, a feature was deemed highly significant if *P* < 0.001, significant if 0.001 ≤ *P* < 0.05, and not significant if *P* ≥ 0.05. For any given pair of (*W*, *G*), only highly significant and significant features were selected for use in the classification process.

### Classification model for predicting tumor necrosis based on MR image features

#### Multi-feature based fuzzy c-means (FCM) clustering

For every combination of (*W*, *G*) and for each given MR image in Table 1, an FCM clustering [48] was performed on the selected vector of features to obtain a fuzzy segmentation map indicating the degree to which each pixel in the tumor AOI (as delineated by a pediatric radiologist) belonged to a necrotic region.

The objective of an FCM clustering is to partition a collection of *q* pixels into *c* overlapping clusters according to *r* features. Such a partition is represented by a matrix *U* = [*u*_*ij*_], where *u*_*ij*_ ∈ [0, 1] is the membership value of pixel *i* to cluster *j* (i.e., the extent to which pixel *i* belongs to cluster *j*), where 1 ≤ *i* ≤ *q* and 1 ≤ *j* ≤ *c*. Let *X* be the matrix, of dimension *q* × *r*, containing *r* features for each of the *q* pixels. In order to find an optimal partition *U*, an objective function is defined as

$$\sum_{j=1}^{c}\sum_{i=1}^{q}{\left(u_{ij}\right)}^{m}{\left\|X_{i}-v_{j}\right\|}^{2}$$ (1)

where *m* is the weighting exponent controlling the degree of fuzziness of the membership function, *X*_*i*_ is the feature vector (of size *r*) for pixel *i*, *v*_*j*_ is the center of cluster *j*, and || · || is the Euclidean norm. The objective function is then minimized by an iterative process, in which the cluster centers and the partition are updated as

$$v_{j}=\frac{\sum_{i=1}^{q}{\left(u_{ij}\right)}^{m}X_{i}}{\sum_{i=1}^{q}{\left(u_{ij}\right)}^{m}}$$ (2)

and

$$u_{ij}=\frac{1}{\sum_{k=1}^{c}{\left(\frac{{\left\|X_{i}-v_{j}\right\|}^{2}}{{\left\|X_{i}-v_{k}\right\|}^{2}}\right)}^{\frac{1}{m-1}}}$$ (3)

respectively. The iterative process is terminated when the Euclidean norm of the difference in *V* = [*v*_*j*_] between two consecutive iterations is less than a sensitivity threshold *ε*.

In the present study, the number of clusters was *c* = 2 (i.e., necrotic and viable tumor regions), the weighting exponent for fuzziness was *m* = 2, and the sensitivity threshold was *ε* = 10^−5^. Without loss of generality, let *u*_*i*1_ and *u*_*i*2_ be the membership values of pixel *i* to necrotic and viable tumor regions, respectively. Given the primary interest was to identify tumor necrosis, for simplicity of notation, *u*_*i*2_ would be omitted, and *u*_*i*_ would be used in place of *u*_*i*1_ hereafter.

#### Weighted majority rule

For every given pair of (*W*, *G*), the individual fuzzy segmentation maps, each of which was computed from a distinct MR image in Table 1, were combined by using a weighted majority ruling to yield a final binary segmentation map indicating whether a pixel in the tumor AOI belonged to necrotic or viable regions. In a weighted majority rule, for a given pixel, if the sum of its weighted membership values to tumor necrosis in the fuzzy segmentation maps being combined was greater than half of the total number of maps, the pixel was then classified as necrotic in the final binary segmentation map. Different weight factors were used to adjust the relative contributions of the individual fuzzy segmentation maps in computing the final binary segmentation map.

Formally, let *s* be the number of fuzzy segmentation maps to be combined. Let *u*_*i*_^*k*^ denote *u*_*i*_ in fuzzy segmentation map *k*, where 1 ≤ *k* ≤ *s*. A binary membership value *y*_*i*_ c∈ {0, 1} is defined for each pixel *i*, where *y*_*i*_ = 1 and *y*_*i*_ = 0 indicate necrotic and viable tumor, respectively. According to a weighted majority ruling, for a given pixel *i*, if $\sum_{k=1}^{s}w_{k}u_{i}^{k}>s/2$, then *y*_*i*_ = 1; otherwise, *y*_*i*_ = 0, where *w*_*k*_ is the weight specifying the relative influence of each fuzzy segmentation map *k*, *k* ∈ [0, *s*], and *w*_*k*_ satisfies $\sum_{k=1}^{s}w_{k}=1$.

#### Weight optimization

The notations introduced earlier were extended to each of *n* different patients as follows. For a patient *ℓ*, where 1 < *ℓ* ≤ *n*, let *q*^*ℓ*^ be the number of pixels in the tumor AOI, *u*_*i*_^*kℓ*^ be the membership value of pixel *i* in fuzzy segmentation map *k*, and *y*_*i*_^*ℓ*^ be the binary membership value of pixel *i*. Let *N*_hist_^*ℓ*^ denote the “gold-standard” reference value of tumor necrosis estimated by means of histopathology. Let *N*_AOI_^*ℓ*^ be the predicted tumor necrosis (in percentage of the tumor AOI), which could be expressed as $\sum_{i=1}^{q^{l}}y_{i}^{l}\cdot 100/q^{l}$. The absolute error was defined as the absolute difference between *N*_AOI_^*ℓ*^ and *N*_hist_^*ℓ*^. In search of a set of optimal weights *w*_*k*_ for distinguishing necrosis from viable tumor, two different objective functions were investigated:

$$f_{\mathrm{avg}}=\frac{1}{n}\sum_{l=1}^{n}|N_{\mathrm{AOI}}{}^{l}-N_{\mathrm{hist}}{}^{l}|$$ (4)

$$f_{\max}=\underset{l}{\max}|N_{\mathrm{AOI}}{}^{l}-N_{\mathrm{hist}}{}^{l}|$$ (5)

Eqs (4) and (5) represent the mean and maximum absolute errors of *n* patients; they would be called min-avg and min-max optimization measures, respectively.

For every given pair of (*W*, *G*), optimal weights *w*_*k*_ were computed using min-avg and min-max optimization approaches, respectively, for each of the following different subsets of MR images (referred to as MRI subsets for brevity):

- CONV (*s* = 3),
- DW (*s* = 1),
- DCE-q (*s* = 4),
- DW + DCE-q (*s* = 5),
- CONV + DW (*s* = 4),
- CONV + DCE-q (*s* = 7),
- CONV + DW + DCE-q (*s* = 8), and
- CONV + DW + DCE-q + DCE-s (*s* = 11),

where *s* denotes the number of distinct MR or derived parametric images in a given MRI subset. The optimization process was carried out in a discrete fashion using a “brute-force” method. Each weight *w*_*k*_ was assumed to be a discrete variable–that is, taking on the discrete values in the closed range of [0, *s*] with a step size *t*. Formally, *w*_*k*_ ∈ {*zt* | *z* ∈ ℤ^≥^, 0 ≤ *zt* ≤ *s*}. Depending on the size *s* of a given MRI subset, a step size *t* was reasonably chosen from [0.01, 1] such that the set of optimal weights {*w*_*k*_ | 1 ≤ *k* ≤ *s*} could be found within reasonable time by iterating through the discrete set of all possible permutations of candidate weights for which $\sum_{k=1}^{s}w_{k}=1$ held true.

For each given combination of weight optimization method (i.e., min-avg or min-max) and MRI subset (e.g., CONV + DW + DCE-q), the optimal value pair of (*W*, *G*) was one that resulted in the lowest value of the objective function (i.e., *f*_avg_ or *f*_max_), and its associated set of optimal weights *w*_*k*_ was subsequently used in the classification model to generate results for further analysis.

### Dice and Szymkiewicz–Simpson coefficients

Two quantitative measures–namely, Dice [49] and Szymkiewicz–Simpson (or overlap) [50] coefficients–were computed to characterize the similarity in spatial distribution between tumor necrosis estimated by histopathology, through a deep learning classification model, and that estimated using MR images. For a given patient *ℓ*, let *A*_nec_^*ℓ*^ denote the set of pixels in the tumor AOI_hist_ belonging to necrosis, and *B*_nec_^*ℓ*^ be the set of necrotic pixels in the tumor AOI. The Dice and overlap coefficients are defined as 2|*A*_nec_^*ℓ*^ ∩ *B*_nec_^*ℓ*^| / |*A*_nec_^*ℓ*^| + |*B*_nec_^*ℓ*^| and |*A*_nec_^*ℓ*^ ∩ *B*_nec_^*ℓ*^| / min(|*A*_nec_^*ℓ*^|, |*B*_nec_^*ℓ*^|), respectively. Both coefficients range between zero and one, with values closer to one indicating a higher degree of similarity.

Note that the Dice coefficient is insensitive to the different sizes of the sets (i.e., *A*_nec_^*ℓ*^ and *B*_nec_^*ℓ*^) being compared. This is reflected by the use of the total size of the two sets as the denominator in the definition for the Dice coefficient. This size-insensitive property is a major drawback of utilizing the Dice coefficient, especially when the two sets being compared have different sizes, which happened to be the case in the present study; more details would be provided in the Discussion section. In contrast, the overlap coefficient takes into account the uneven sizes of the two sets being compared. In fact, through the use of the size of the smaller set as the denominator in its definition, the overlap coefficient indicates the extent to which one set contains the other (i.e., the overlap coefficient is one when one set is completely within the other). Both the Dice and overlap coefficients were considered herein as they would provide different valuable insights in assessing the similarity of tumor necrosis between the AOI_hist_ and AOI.

### Evaluation of tumor necrosis based on tumor VOI

For every combination of weight optimization method and MRI subset, the classification model established with the previously determined set of optimal weights *w*_*k*_ was used to compute tumor necrosis from the entire tumor VOI. Let *N*_VOI_^*ℓ*^ denote the percentage of necrosis computed from the tumor VOI. The VOI-based tumor necrosis was then compared to that of the tumor AOI and the estimation reported by histopathology, respectively; in particular, their actual differences *N*_VOI_^*ℓ*^–*N*_AOI_^*ℓ*^ and *N*_VOI_^*ℓ*^–*N*_hist_^*ℓ*^ were calculated.

### Sample size calculation

A power analysis was performed a priori, based on which a sample size of 24 prospective patients, assuming that half of them would be responsive to treatment, would provide 80% power to detect an odds ratio of at least 4.5 per standard deviation of a normally distributed predictive feature. Unfortunately, the desired number of prospective subjects was not achieved by the end of the study (February 2020). The study was initially closed to patient enrollment because of slow accrual due to referral and intuitional considerations, and the accrual was discontinued towards the end of the study because of the pandemic.

### Statistical analysis

Results are presented as mean ± standard error of the mean. Each pairwise statistical comparison was performed using one-way ANOVA [47], and *P* < 0.05 was considered statistically significant.

## Results

The present study enrolled 15 consecutive patients (7 male and 8 female) diagnosed with high-grade appendicular osteosarcoma in a single-center observational study between November 1, 2016 and April 7, 2019 (see Table 3 for a summary of the demographic data of the patients). The patients ranged in age from 10 to 20 years (median = 13 years). Bone tumors were located in the femur in five cases, in the tibia in four cases, in the humerus in three cases, and in the fibula and radius in one case each. Two patients withdrew and one patient became ineligible to proceed mid-study. Two patients were excluded due to lack of pre-surgical (week-10) imaging. In the end, a total of *n* = 10 patients were considered for further imaging data processing and analysis. In addition, for three of the *n* = 10 patients, a DCE-MRI sequence was either not acquired or invalid due to logistical or image acquisition issues.

**Table 3:** Demographic information for patients in the study (patients included in the imaging data analysis are highlighted).

Patient no.	Age	Sex	Ethnicity	Tumor location
1	20	Male	White	Femur
2	13	Female	Hispanic	Radius
3	12	Male	White	Fibula
4	14	Female	Black	Femur
5	16	Male	Hispanic	Humerus
6	11	Female	White	Femur
7	10	Female	Black	Femur
8	12	Female	White	Tibia
9	16	Male	Hispanic	Tibia
10	12	Female	Black	Tibia
11	13	Female	Hispanic	Humerus
12	17	Male	Black	Tibia
13	13	Male	White	Femur
14	11	Female	White	Humerus
15	16	Male	Black	Femur

**Fig 2:**
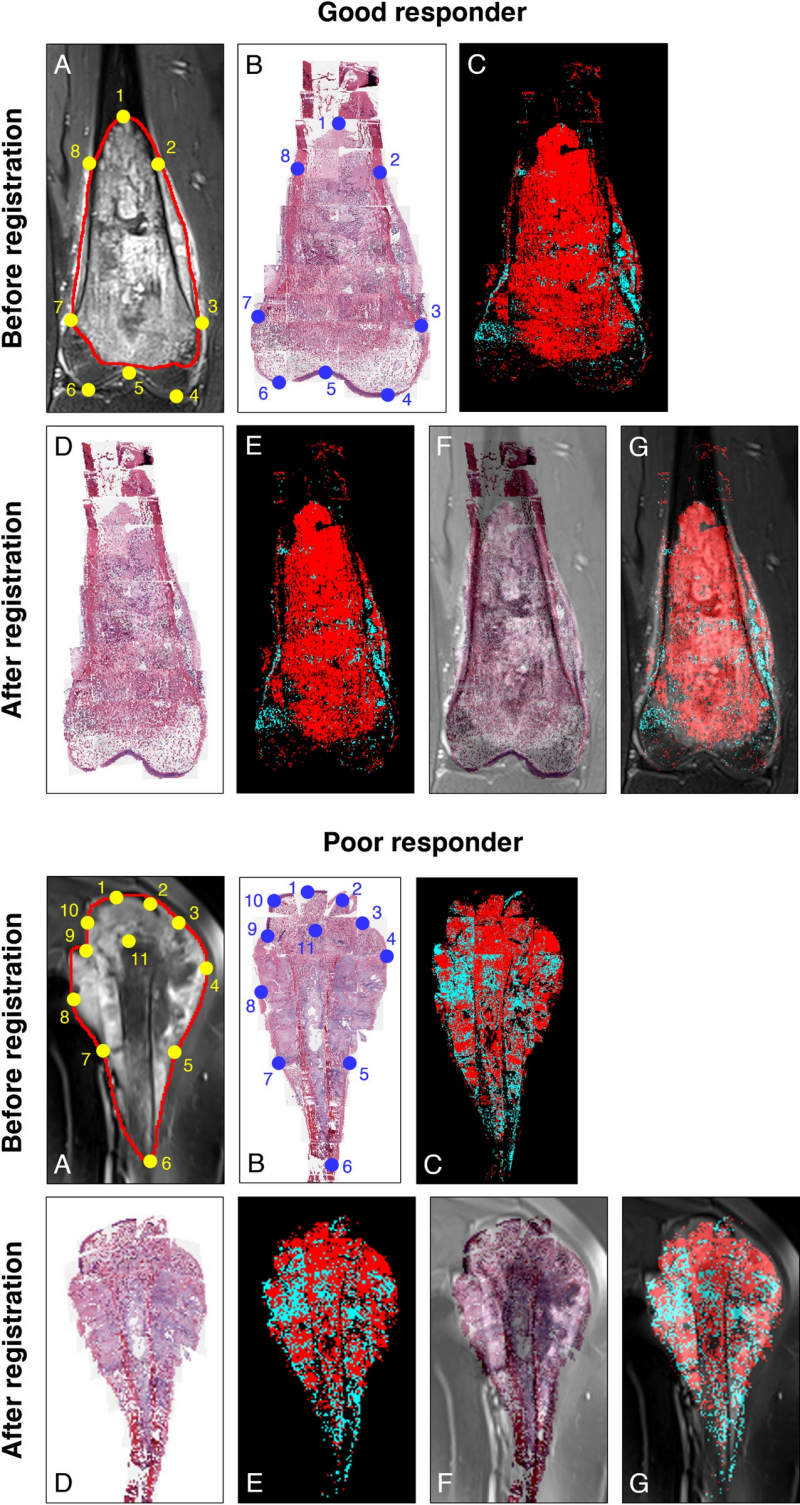
Co-registration of histologic and MR images of osteosarcoma in POI through control point mapping. Representative images are shown for a good-responding patient (*N*_hist_^*ℓ*^ ≥ 90%) and a poor-responding patient (*N*_hist_^*ℓ*^ < 90%). (A) Post-contrast T1-weighted MR image, with the boundary of the tumor AOI drawn in red. (B) Histologic section image (reconstructed from individual whole slide images) prior to image registration, and (C) its corresponding histologic tumor viability map (wherein necrotic and viable tumor regions are indicated in red and blue, respectively) computed by a deep learning classification model. Each pair of defined control points is represented by a yellow dot (in MR image) and a blue dot (in histologic image) labeled with the same number. (D) Histologic image and (E) its associated tumor viability map after being co-registered to MR image. (F) Average intensity projection of MR image fused with histologic image. (G) Average intensity projection of MR image fused with histologic tumor viability map.

The process of correlating histopathology and MRI begins by registering a primary histologic section image to an MR image in the POI. Fig 2 showcases, using the instances of two different patients (i.e., a good responder with *N*_hist_^*ℓ*^ ≥ 90% and a poor responder with *N*_hist_^*ℓ*^ < 90%), the histologic section image and the MR image (post-contrast T1-weighted) in the POI before and after their co-registration. Control points are defined along the boundary of tumor mass and neighboring anatomical landmarks such as the epiphysis and growth plate. As visually verified by a pathologist and a pediatric radiologist, based on the overlay of the MR image and its co-registered histologic image (Fig 2F and 2G), the contour of the tumor in the MR image appears well aligned with that in the histologic image.

**Fig 3:**
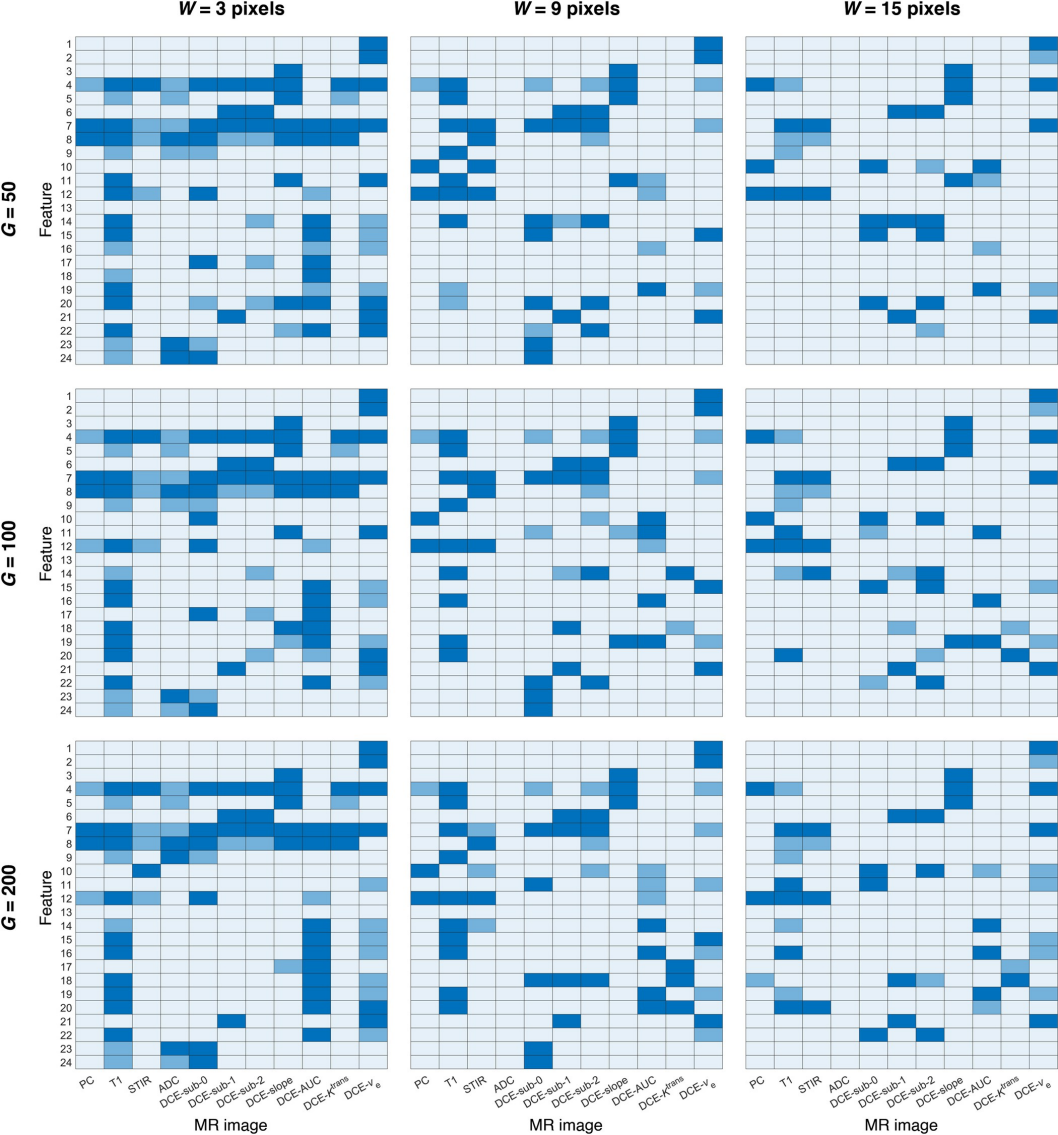
Statistical significance of difference in MR image features between histologic necrosis and viable tumor. *W* and *G* denote the window size and the number of gray levels, respectively. Features are identified by their numbering in Table 2. Light blue indicates *P* < 0.001 (highly significant), medium blue indicates 0.001 ≤ *P* < 0.05 (significant), and dark blue indicates *P* ≥ 0.05 (not significant).

The registration of a histologic section image to an MR image in the POI allows for the identification of histological necrotic and viable tumor regions in the MR image. In search of the best *MR image features that differentiate between necrotic and viable tumor regions*, statistical and Haralick texture features are computed from various MR images using different pixel-centered window sizes and numbers of gray levels. In Fig 3, a three-color heatmap is used to represent the significance levels of the features in distinguishing necrosis from viable tumor. In general, as the size of the window increases, more features are identified as statistically significant (*P* < 0.05) in differentiating between viable and necrotic tumor regions. Regardless of the number of gray levels, when the size of the window increases from 3 to 15 pixels, the number of significant features increases for each given MR image by 2 ± 4 (minimum = 1, maximum = 8, median = 3). The only exceptions are PC, STIR, and DCE-sub-2, for which the number of significant features either remains the same or decreases by one as the size of the window increases. The set of significant features tends to differ considerably from one MR image type to another, as can be observed in Fig 3. For instance, for T1, most Haralick texture features are insignificant at *W* = 3 pixels, but become significant at *W* = 15 pixels. In the contrary, for PC and STIR, most Haralick texture features are significant at *W* = 3 pixels, and remain so as *W* increases to 15 pixels. On the other hand, changing the number of gray levels does not have a noticeable effect on the number of significant features. At any given window size, the number of significant features remains the same for most MR images as the number of gray levels increases from 50 to 200. For any given MR image, the variation in the number of significant features is 0 ± 1 (minimum = -2, maximum = 2, median = 0) as the number of gray levels changes.

**Fig 4:**
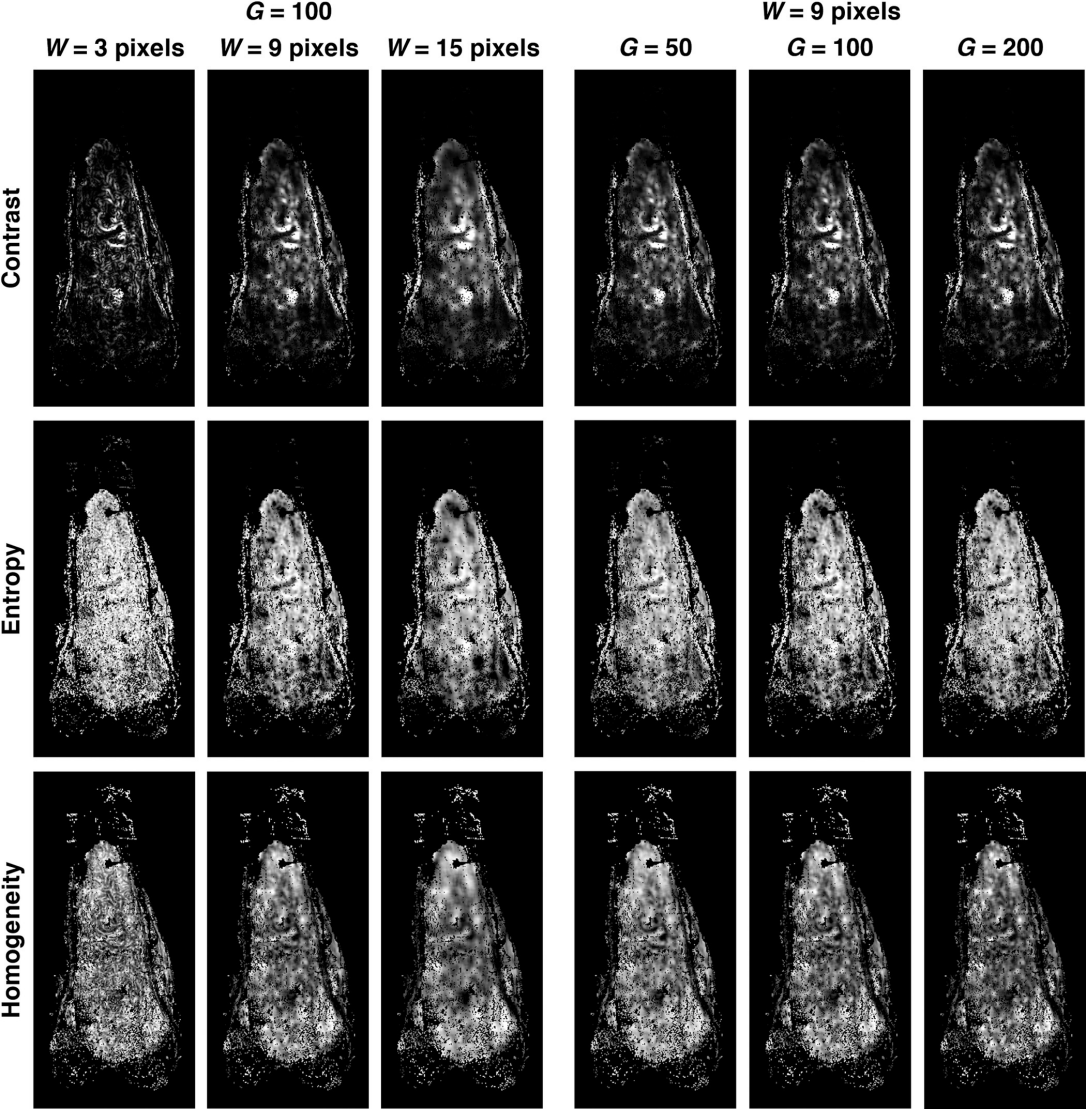
Effects of window size *W* and number of gray levels *G* on Haralick texture features. Images show the differences in texture features, such as contrast, entropy, and homogeneity, computed for the tumor AOI_hist_ in the post-contrast T1-weighted MR image as window size *W* varies (from 3 to 9 to 15 pixels) while keeping the number of gray levels constant at *G* = 100. No visible differences can be observed in the texture features when the number of gray levels *G* changes from 50 to 100 to 200 while the window size remains constant at *W* = 9 pixels.

Fig 4 shows, through the examples of three Haralick texture features (i.e., contrast, entropy, and homogeneity), the effect of using different window sizes and numbers of gray levels when computing the features. When the number of gray levels is kept constant (at 100), there are large regions of relatively uniform intensity in the feature image computed using a window size of *W* = 15 pixels, and the texture appears quite different from the speckled pattern observed in the feature image calculated using *W* = 3 pixels. Generally, a larger window results in a decrease in the spatial resolution of the feature computed, but affords a macroscale spatial depiction of the feature–a representation not possible when using a smaller window size, at which the fine textures at the microscale are captured in detail. In contrast, increasing the number of gray levels from 50 to 100 have no visible effect on the Haralick texture features computed from the MR images. As demonstrated in Fig 4, the feature images appear almost similar regardless of the number of the gray levels used when the size of the window is constant (at 9 pixels). This implies that the numbers of gray levels *G* = 50, 100, and 200 are similarly adequate in resolving intensity variations in the given MR images when computing the GLCM.

**Fig 5:**
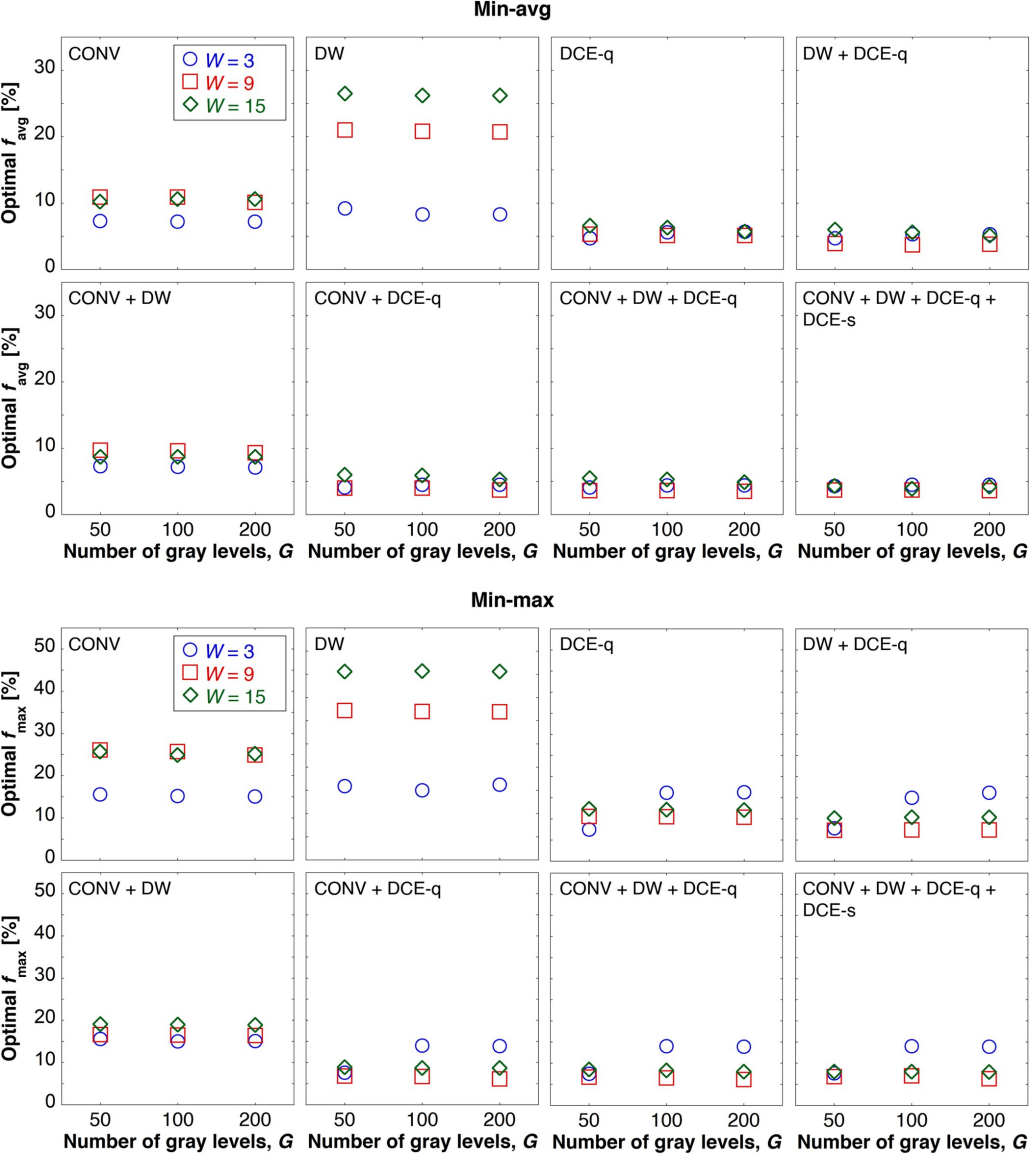
Optimal values of objective function for different combinations of weight optimization methods and MRI subsets. Optimal values of objective functions *f*_avg_ and *f*_max_ represent the minimized mean absolute error and the minimized maximum absolute error, respectively, obtained in the weight optimization process.

For each predefined MRI subset, the optimal weight factors, which indicate the relative contributions of each distinct MR image in the given MRI subset for computing the tumor viability map, are determined using two optimization methods (i.e., min-avg and min-max) for different value pairs of window size and number of gray levels. Fig 5 shows the results from the weight optimization procedure–specifically, the optimal value of the objective function *f*_avg_ or *f*_max_ (depending on which weight optimization measure is used) for each different combination of MRI subset, window size, and number of gray levels. In consistency with the previous observation on the noticeable impact of changing the window size on MR image features as opposed to the negligible effect of the number of gray levels, when the number of gray levels changes from 50 to 200, the resulting optimal values of the objective function do not deviate as considerably as those when the window size changes from 3 to 15 pixels. Nonetheless, for subsequent data processing and analysis, the best settings of window size and number of gray levels–that is, those that yield the lowest optimal value of the objective function–are selected for each MRI subset. For either weight optimization method, *W* = 3 pixels has been shown to be the optimal window size for CONV, DW, and CONV + DW, while *W* = 9 pixels is optimal for all other MRI subsets. For both weight optimization methods, *G* = 50 is the optimal number of gray levels for DCE-q, *G* = 100 for DW and DW + DCE-q, and *G* = 200 for all the others.

**Fig 6:**
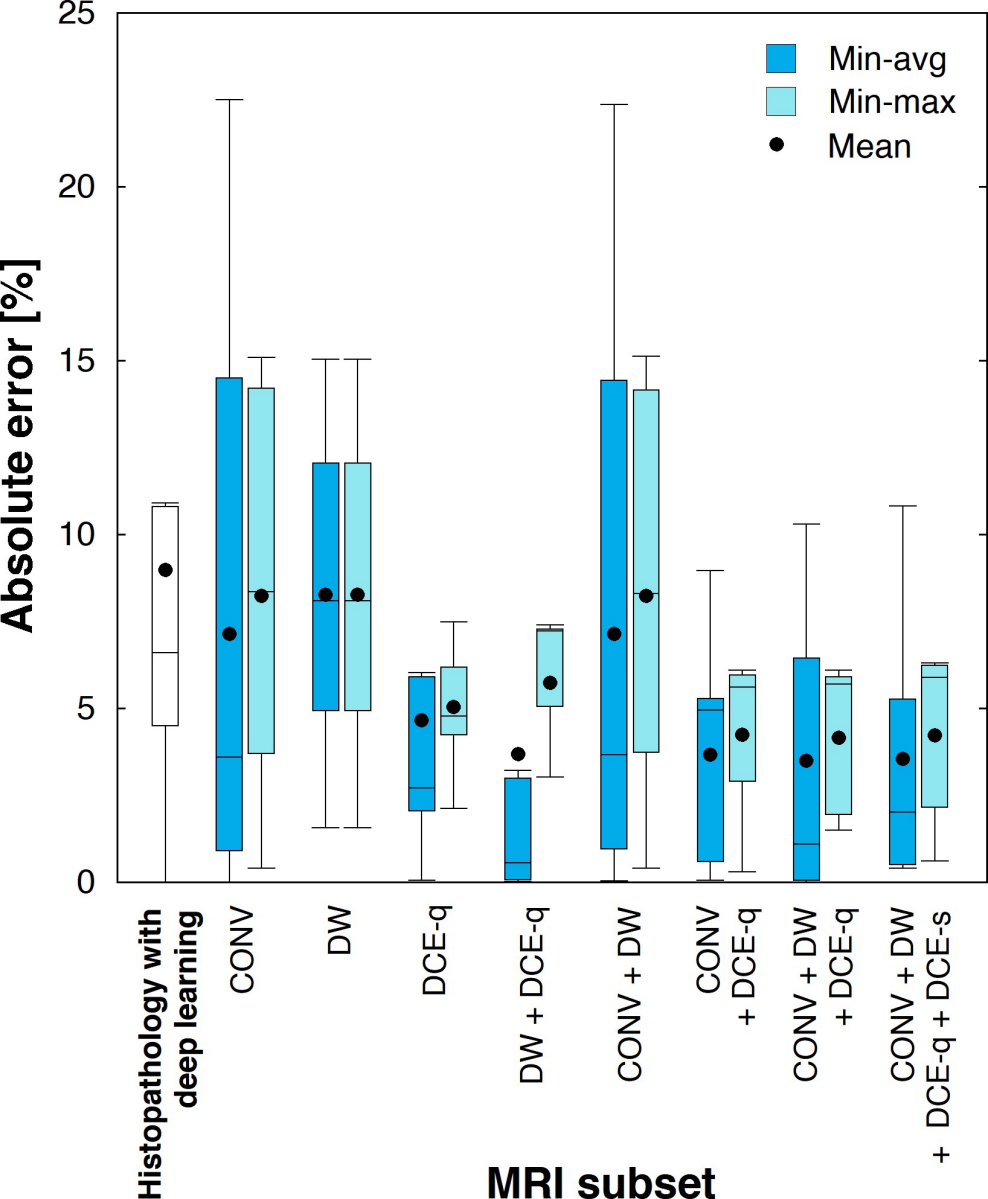
Absolute error in tumor necrosis estimated using various combinations of weight optimization methods and MRI subsets. The leftmost box represents the absolute error in histologic tumor necrosis estimated from primary histologic section images using a previously established deep learning classification method.

At those optimal settings of (*W*, *G*), a comparison is made between tumor necrosis estimated from MR images and that evaluated by a pathologist. Fig 6 shows the absolute error in tumor necrosis estimated from various MRI subsets using either min-avg or min-max optimization approaches. Overall, in comparison to min-avg optimization, min-max yields a set of optimal weights that result in tumor necrosis predictions with a higher absolute error and a lower variability (indicated by a smaller standard error of the mean). For any given MRI subset, there is no statistically significant difference in the absolute error between the two weight optimization methods. For min-max optimization, a statistically significant difference (*P* ≤ 0.03) is observed in the absolute error between i) CONV and CONV + DCE-q, ii) CONV and CONV + DW + DCE-q, iii) CONV and CONV + DW + DCE-q + DCE-s, iv) CONV + DW and CONV + DCE-q, v) CONV + DW and CONV + DW + DCE-q, and vi) CONV + DW and CONV + DW + DCE-q + DCE-s. However, no statistically significant difference (*P* > 0.05) is observed in the absolute error between i) CONV and CONV + DW, ii) CONV + DCE-q and CONV + DW + DCE-q, and iii) CONV + DW + DCE-q and CONV + DW + DCE-q + DCE-s. The main implications of these statistical results are two-fold. First, adding DCE-q to CONV significantly improves the accuracy of the resulting tumor necrosis prediction, whereas adding DW does not. Secondly, the accuracy of the estimated tumor necrosis does not improve by adding DCE-s to CONV + DW + DCE-q. As seen in Fig 6, CONV + DCE-q, CONV + DW + DCE-q and CONV + DW + DCE-q + DCE-s are the three MRI subsets that yield the best estimations of tumor necrosis with the highest accuracies (with reference to histopathology). For instance, the absolute error for CONV + DW + DCE-q is 4.1 ± 0.8% (*n* = 7). On the other hand, CONV, DW, and CONV + DW produce the worst results with the lowest accuracies. In the case of CONV, an absolute error of 8.2 ± 1.7% (*n* = 10) is observed. For min-avg optimization, no statistically significant difference (*P* > 0.05) is found in the absolute error between any two given MRI subsets.

**Fig 7:**
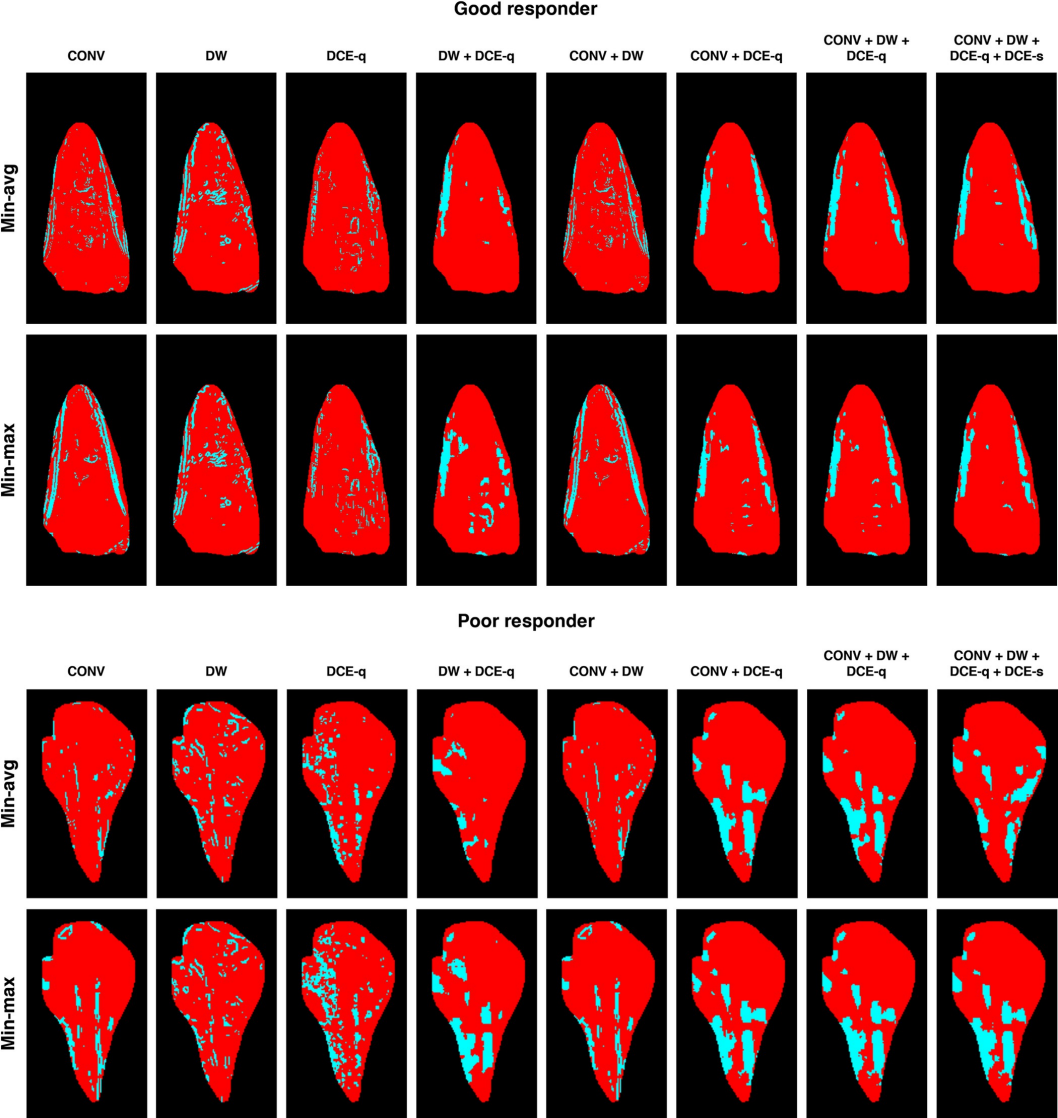
Viability mapping of tumor AOI computed using different combinations of weight optimization methods and MRI subsets. Tumor viability maps are shown for a good-responding patient (*N*_hist_^*ℓ*^ ≥ 90%) and a poor-responding patient (*N*_hist_^*ℓ*^ < 90%). Necrotic and viable tumor regions are represented in red and blue, respectively.

**Fig 8:**
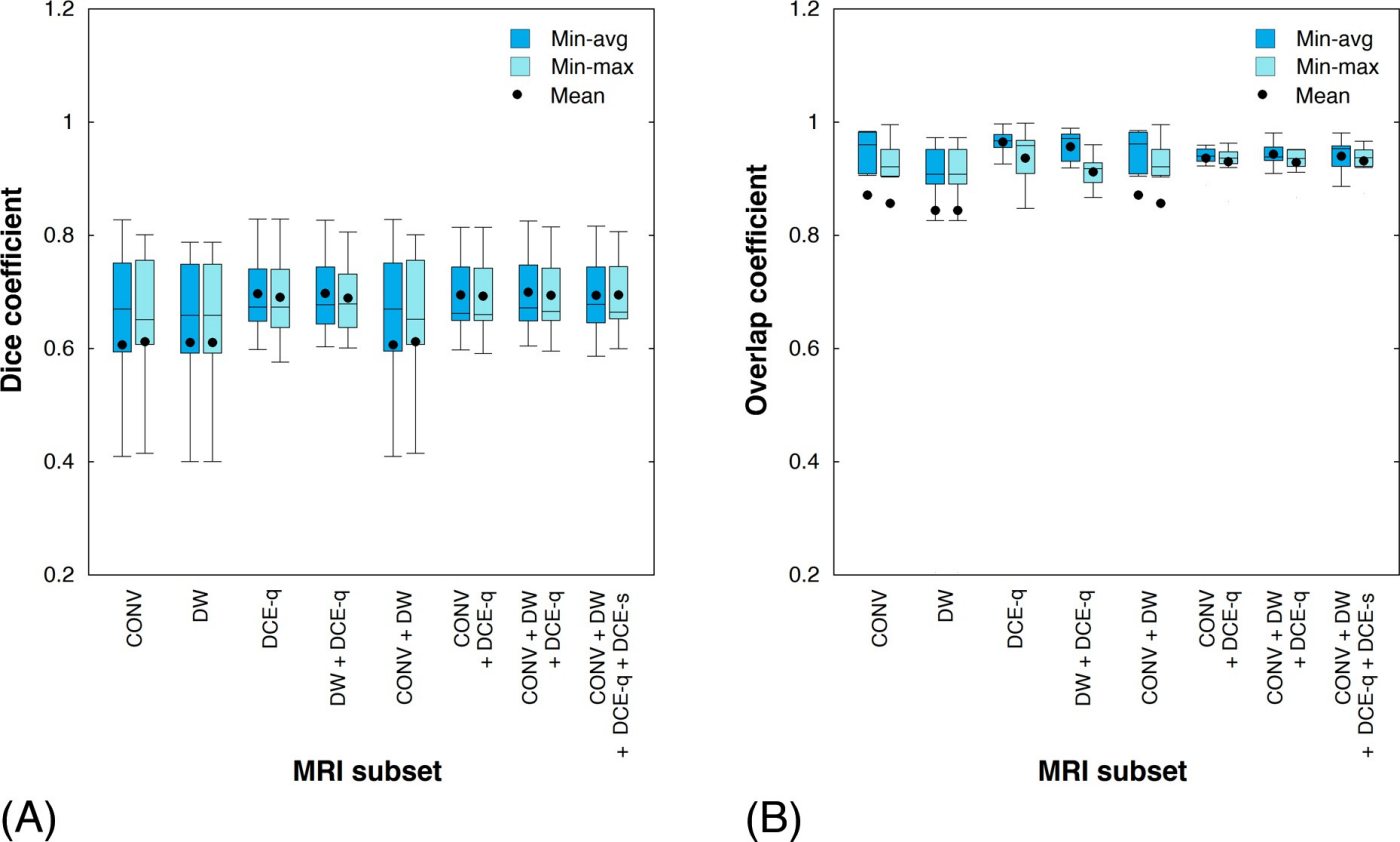
Measures of similarity of necrotic tumor region between tumor viability map computed by histopathology and that estimated from various MRI subsets. (A) Dice coefficient. (B) Overlap coefficient.

Fig 7 illustrates, through the instances of the same two patients as in Fig 2, the viability maps for the tumor AOI computed from different MRI subsets using either of the two weight optimization approaches. Compared with histologic tumor viability maps (Fig 2E), the tumor viability maps estimated from MR images appear quite similar (if not completely the same) in their overall spatial distribution of necrotic and viable tumor regions. The Dice and overlap coefficients are employed for measuring the said similarity for each MRI subset (Fig 8). For both weight optimization methods, no statistically significant difference in either the Dice or overlap coefficients is found between any two given MRI subsets. Likewise, for any given MRI subset, neither of the two coefficients are significantly different between the two weight optimization methods. Yet, the general profile of the computed measures is similar to that previously observed in the absolute errors of tumor necrosis estimations. Particularly, CONV + DCE-q, CONV + DW + DCE-q, and CONV + DW + DCE-q + DCE-s are among the MRI subsets that yield the highest similarity. For instance, the Dice and overlap coefficients are 0.70 ± 0.03 and 0.94 ± 0.01 (*n* = 7), respectively, for CONV + DW + DCE-q (using min-avg optimization). On the contrary, CONV, DW, and CONV + DW produce the tumor viability maps with the least similarity to that estimated by histopathology. In the case of CONV, the Dice and overlap coefficients are 0.61 ± 0.07 and 0.87 ± 0.09 (*n* = 10), respectively. As expected, the Dice coefficient is significantly lower than the overlap coefficient. This implies that the sizes of *A*_nec_^*ℓ*^ and *B*_nec_^*ℓ*^ are indeed quite different. But, as indicated by a relatively high overlap coefficient, one set is more or less contained within the other.

**Fig 9:**
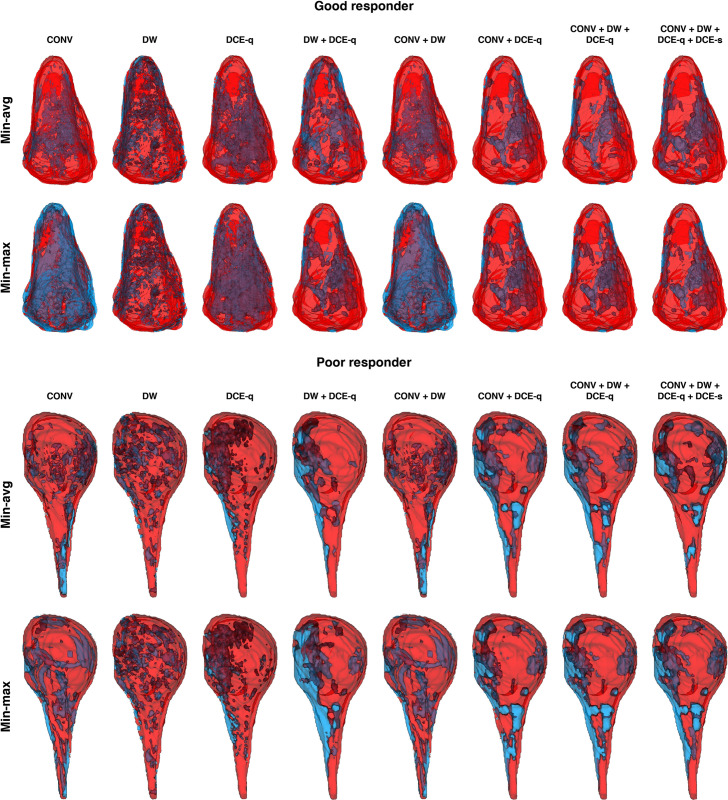
Viability maps for tumor VOI estimated using different combinations of weight optimization methods and MRI subsets. Three-dimensional tumor viability maps are shown in the coronal orientation for a good responder (*N*_hist_^*ℓ*^ ≥ 90%) and a poor responder (*N*_hist_^*ℓ*^ < 90%).Necrotic and viable tumor regions are represented in red and blue, respectively.

**Fig 10:**
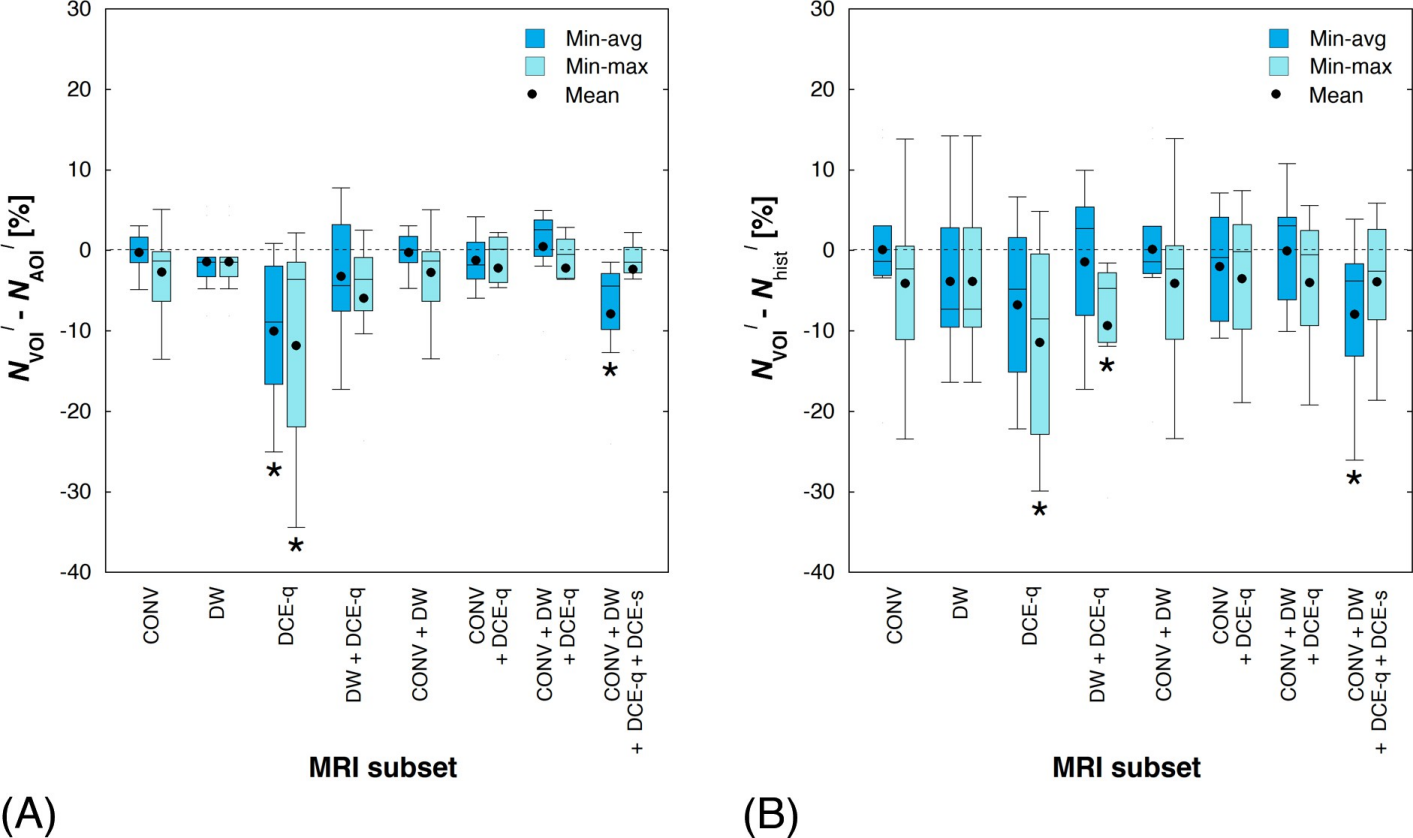
Comparison of volume- and single plane-based estimations of tumor necrosis. (A) Actual difference in tumor necrosis estimation between that of VOI and AOI (i.e., *N*_VOI_^*ℓ*^–*N*_AOI_^*ℓ*^). (B) Actual difference between VOI-based and histopathologic tumor necrosis estimations (i.e., *N*_VOI_^*ℓ*^–*N*_hist_^*ℓ*^). An asterisk below a box indicates that the associated actual difference is statistically significant (*P* < 0.05).

Finally, the MRI-based classification model, established earlier by using a correlation between histologic and MR images in the POI, was used to estimate tumor necrosis for the entire VOI. Fig 9 shows the three-dimensional viability maps for the tumor VOI computed from various MRI subsets using either of the two weight optimization methods. Through the coronal orientation view of the three-dimensional tumor viability map (i.e., in the same orthogonal direction of the POI), it appears that, for a given patient, the overall distribution of tumor necrosis, as well as its relative proportion with respect to viable tumor, could be quite different from those inferred from the POI. In Fig 10, the amount of tumor necrosis estimated for the VOI is compared to that for the AOI and the estimation provided by a pathologist. Although a statistical significance is not seen across the board, a clear overall negative inclination is observed in the actual differences between *N*_VOI_^*ℓ*^ and *N*_AOI_^*ℓ*^ (or between *N*_VOI_^*ℓ*^ and *N*_hist_^*ℓ*^)–that is, the volume-based tumor necrosis estimations tend to be lower than those evaluated based on a single tissue section or MRI slice.

## Discussion

The current study set out to find a reliable and accurate classification model for differentiating necrosis from viable tumor in patients with high-grade osteosarcoma using MR images. We used a previously developed machine learning tool to identify histologic necrosis versus viable tumor in resected osteosarcoma. Through histologic-MR image co-registration, we built a model to extract MR image features to identify necrosis and viable tumor. Without prior knowledge of what statistical and textural MR image features sufficiently characterize histologic necrosis and viable tumor, as well as the scales at which the features properly reflect the qualities of the two regions of interest, different features and analysis parameters (such as pixel-centered window size and number of gray levels) were examined in search of the best features and parameter settings. In the process of establishing a classification model, MR images of different modalities and their various combinations were taken into consideration. An optimal weighted majority ruling was sought such that the individual MR images were combined in an optimal ratio to yield the best possible prediction of tumor necrosis given a specific error minimization criterion.

The main findings were as follows. MR image features shown to be significant in distinguishing necrosis from viable tumor were different depending on the modality or type of the given MR image (Fig 3). The scales at which the features optimally indicated necrotic and viable tumor were different as well depending on the MR image type (Fig 5). Conventional MR images (i.e., PC, T1, and STIR) alone were sufficiently capable of differentiating necrosis from viable tumor, with an accuracy averaging above 90%. Conventional MR images were equally effective as ADC in distinguishing necrotic from viable tumor regions. The accuracy of tumor necrosis prediction by conventional MR images improved when DCE-q (i.e., DCE-slope, DCE-AUC, DCE-*K*^trans^, and DCE-*v*_e_) was added into consideration. The same did not occur when adding ADC. With respect to DCE-MRI, no additional benefit was found in adding DCE-s (i.e., subtraction images) when DCE-q was already included.

### Conventional MRI

The current findings as regards conventional MRI are encouraging from the perspective of a radiologist, given that conventional MRI is commonly performed and readily available at diagnosis and mid-treatment. In fact, it is not surprising that conventional MR images could be informative (to a certain extent) in approximating the degree of tumor necrosis. Researchers have shown that visual interpretations of conventional MR images are indeed possible for the prognosis of osteosarcoma [10, 51, 52]. First, intraosseous (intramedullary) tumor is evident on T1-weighted MR images as areas of low signal intensity. Areas of high T1 signal intensity within a lesion typically represent areas of hemorrhage, whereas areas of low T1 signal intensity within a lesion usually correspond to areas of bone formation (dense mineralization). Post-contrast T1-weighted MR images (with fat suppression), through a contrast enhancement of well-vascularized tissues, allow the differentiation of vascularized tumoral areas (e.g., viable tumor, granulated or fibrous tissue) and non-vascularized tumoral areas (e.g., liquefaction necrosis). However, these images do not distinguish viable tumor from immature vascularized granulation tissue, fibrous tissue, neovascularity in necrotic areas, and reactive hyperemia. In other words, static post-contrast T1-weighted MR images are highly sensitive but lack of specificity, which could lead to an overestimation of the amount of residual viable tumor. STIR imaging is commonly used for detecting abnormal fluid content within tissues such as tumor, edema, and infection [53, 54]. A high STIR signal is typically seen within most tumors due to their increased water content. A low STIR signal within tumor usually indicates bone formation (dense mineralization).

MRI sequences acquired at diagnosis usually comprise non-contrast T1-weighted, post-contrast fat-saturated T1 weighted, and STIR. Dynamic contrast-enhanced MRI and DWI are typically not indicated for routine clinical use. Hence, while these advanced MRI techniques are important additional prognostic factors predictive of tumor necrosis, it was imperative for the current study to examine the value of conventional MRI sequences (alone or combined with other sequences) in assessing tumor response to chemotherapy. A classification model was created to “interpret” conventional MR images of osteosarcoma, and it was shown that a reasonable estimate of tumor necrosis could be obtained through the model.

### DCE-MRI

The use of multi-modal MR images, especially advanced MRI sequences, was demonstrated herein to be critical in achieving the greatest accuracy in prediction of tumor necrosis. Specifically, DCE-MRI sequence and its derived parametric images were proven in the current study to play a significant role in differentiating necrosis from viable tumor. This observation is consistent with various prior results on the capacity of DCE-MRI in evaluating musculoskeletal sarcomas, from quantifying tumor necrosis to identifying good responders. For instance, semi-quantitative parametric images (i.e., DCE-s, DCE-slope, and DCE-AUC) are important for detecting early and progressive enhancing structures in tumors. Other studies demonstrated an excellent correlation between early enhancing lesions, viable tumors, and poor chemotherapeutic (histologic) response [8, 27, 28, 43, 55, 56]. The degree of viability tends to vary in different parts of tumor, and DCE-MRI exhibits the potential of correctly identifying viable tumor regions by showing different local enhancement patterns. Particularly, as seen in [27, 28, 57], early and rapid enhancement patterns in DCE-MRI sequences could be used to identify viable tumor (although early enhancing regions might also include normal physes and arteries). Furthermore, most DCE-MRI studies performed in patients with osteosarcoma demonstrated that a decrease in the slope of the time-intensity curve, tumor blood volume (*v*_e_), and vessel permeability (*K*^trans^) reflects a favorable therapeutic response to chemotherapy. Specifically, as previously shown in [8, 56, 58], a significantly greater reduction in the slope of the time-intensity curve was observed in good responders. A comprehensive meta-analysis of six previous studies (with a total of 66 patients) [59] concluded that a greater than 60% reduction in the slope of the time-intensity curve indicated a good response. Indeed, as later reported in [15], the slope of and the area under the time-intensity curve were significantly different between viable and necrotic tumor regions. On the other hand, only a limited number of studies have investigated the effectiveness of using quantitative kinetic parameters in predicting chemotherapeutic response of osteosarcoma. Nevertheless, significant changes in the values of pharmacokinetic parameters have been observed during and after treatment. In particular, according to [45, 60, 61], significantly lower *K*^trans^ and *v*_e_ were found in good responders than poor responders after chemotherapy. In short, there exists abundant scientific evidence in support of the validity of the classification model in using DCE-MRI to differentiate tumor necrosis and residual viable neoplastic tissue.

It is interesting to note that no significant improvement was obtained in the estimation of tumor necrosis when DCE-s was added to the MRI subset already containing DCE-q. It seems that DCE-s did not contribute any additional information useful for improving the accuracy of tumor necrosis prediction, as DCE-slope and DCE-AUC were likely equivalent to DCE-s in terms of representing critical spatial information of contrast enhancement pattern of tumor.

### DWI and ADC

Unlike DCE-MRI sequences, ADC was shown to be ineffective in improving the accuracy of tumor necrosis estimation by conventional MR images. In fact, ADC produced a tumor necrosis estimation with a similar accuracy as conventional MR images. Nonetheless, the potential of using ADC for identifying tumor necrosis was indisputable, as reinforced by the finding of various image features that were significant in distinguishing necrotic from viable tumor regions (Fig 3). It has been generally agreed in the literature that DWI reflects the tissue cellularity of musculoskeletal tumors, and a lower ADC is observed in more cellular and aggressive tumors due to restricted diffusion of water [23]. According to various studies [13, 14, 16, 18, 21, 22, 26], comparing the ADC before and after neoadjuvant chemotherapy showed that the minimum and mean ADC increased after therapy. It was postulated that tumor necrosis has a significantly different ADC from viable tumor, substantially contributing to the differences observed before and after treatment. Indeed, a significantly higher ADC was observed in tumor necrosis, as compared to viable tumor region [14–16]. Tumor necrosis causes an increase in ADC due to increased cellular permeability and decreased cellularity. Furthermore, significantly [21] or non-significantly [19] higher ADCs in good responders were reported after chemotherapy. In [16], the minimum ADC was found to be significantly higher in good responders than poor responders. In [19], a significant difference in ADC was observed between good and poor responders at mid-course of chemotherapy. Byun et al. [20] showed that a 13% increase of ADC allowed a prediction of good response with a sensitivity, specificity, and accuracy of 83%, 73%, and 78%, respectively. Most recently, a meta-analysis of five prior studies (with a total of 106 patients) [25] confirmed the ability of using DWI to predict response of osteosarcoma to chemotherapy.

### AOI vs. VOI

In spite of its overall statistical insignificance, the degree of tumor necrosis estimated from the VOI was generally less than that estimated from the AOI (Fig 10). This observation suggests that, as some studies have claimed in the past [62–64], the amount of residual viable tumor estimated from the entire tumor volume could be different than that estimated from a single histologic section. In fact, a volumetric evaluation of tumor necrosis may be a better prediction than a histopathological estimation, which is based on a selected section of tumor, chosen by a pathologist and may not accurately represent the entire tumor mass. A further investigation, such as histopathological grading of multiple sections of tumor, is required to confirm the validity of using tumor volume for estimating histologic necrosis. Potential implications of VOI-predicted response may include improved surgical planning as well as more precise prognostic information and precision therapy for patients.

### Histopathology-MRI correlation

In order to establish a correlation between histopathologic necrosis and MR image features, pre-identified regions of histologic tumor necrosis were mapped onto a corresponding MR image in the POI through a co-registration between histologic and MR images. Instead of requiring a pathologist to manually delineate necrotic tumor regions in each primary histologic section, a previously established deep learning classifier [39], which was a result of an extensive evaluation against various models [65, 66], was used for the task and to generate histologic tumor viability maps. This helped to eliminate human errors and bias in the segmentation process. However, it was observed that a significant amount of liquified necrotic tumor tissue could be lost during tissue processing for histopathology. This essentially led to an inaccurate estimation of the actual amount of tumor necrosis for some patients by the deep learning classifier, contributing to the comparatively high absolute errors associated with the deep learning classification method as observed in the results (Fig 6), even though the necrotic tumor regions in each histologic section were indeed correctly identified (as verified by a pathologist). In other words, the histologic tumor viability maps produced by the deep learning classifier were accurate in identifying tumor necrosis, albeit being partial and incomplete. Introspectively, this finding further underscores the relative advantage of using MRI in evaluating tumor necrosis without the concern of artifacts and tissue loss induced by the preparation for histopathology.

Unlike other tumor types, osteosarcomas are commonly heterogenous. Due to the heterogeneous nature of the tumors, it is problematic to correlate changes in image intensity (or other parameters derived from MRI sequences) with tumor necrosis. Texture-based statistical measures [46, 67, 68] are useful for characterizing intra-tumoral heterogeneity and for describing local spatial distributions of signal intensities that are important for identifying a given region of interest such as tumor necrosis. Since different analytic parameters (such as window size and number of gray levels) could affect the calculations of texture features (Fig 4), a parametric investigation was undertaken to determine the optimal values of the parameters. The results show that, depending on the modality and type of a given MR image, different texture features computed with varied analytic parameters could be associated with tumor necrosis (Fig 3).

### Measuring accuracy of tumor viability mapping

To evaluate the similarity in spatial distribution of tumor necrosis between AOI_hist_ and AOI, the Dice and overlap coefficients were both employed. Due to liquefaction of necrotic tissue mass and its subsequent loss during specimen preparation for pathologic examination, it was observed that a histologic tumor section might contain void areas, which were considered as non-tumor by the deep learning classification model (i.e., black areas within the boundary of tumor in Fig 2C). Furthermore, the existence of non-tumor tissue within a tumor mass and possible artifacts resulting from tissue sample preparation (for histology) and image stitching could contribute to additional non-tumor areas within the contour of tumor in a histologic viability map. In contrast, each pixel in the tumor AOI (i.e., within the contour of tumor in an MR image as delineated by a pediatric radiologist) was categorized as either necrotic or viable by the MRI-based classification model. Consequently, the number of necrotic tumor pixels |*A*_nec_^*ℓ*^| in the AOI_hist_ was generally lower than the number of necrotic tumor pixels |*B*_nec_^*ℓ*^| in the AOI, even though their bounding contours of tumor and their necrosis estimations (in percentage of the tumor) might be similar. Thus, the Dice coefficient alone might not be representative of the overlap between the actual histologic tumor necrosis and the tumor necrosis estimated from MR images. This helps to explain the difference between the Dice and overlap coefficients observed in the current study. Namely, the overlap coefficient was relatively high (close to one), indicating that *A*_nec_^*ℓ*^ was roughly a subset of *B*_nec_^*ℓ*^. But, in comparison with the overlap coefficient, the Dice coefficient was generally skewed low, implying that the sizes of *A*_nec_^*ℓ*^ and *B*_nec_^*ℓ*^ were indeed different due to the reasons aforenoted.

### Medical image fusion

Research literature refers to the workflow depicted in Fig 1 as image fusion, which consists of image registration and “fusion” of relevant features from the registered images (see [69–71] for general reviews). In the current study, morphological filters were used for feature detection (i.e., pixel-centered window-based image feature extraction), and it was followed by the utilization of a fuzzy membership function for image feature aggregation (i.e., for combining features from different MRI modalities for accurately identifying tumor necrosis). The selection of feature extraction method and aggregation function that result in an optimal image fusion remains an open problem and is indeed often dependent upon given medical applications (e.g., organs and imaging modalities under study). Partially due to a small sample size (*n* ≤ 10), the authors chose to create a relatively simple classification model based on fuzzy *c*-means clustering and weighted majority ruling. Nevertheless, the model was proven sufficient in illustrating the feasibility of predicting tumor necrosis by soft clustering on MR image features, following which a weighted majority ruling was performed to obtain a hard two-class prediction (i.e., necrosis and viable tumor).

### Other limitations and future directions

The current results show, by and large, minimizing the average absolute error, when determining the optimal weights for combining MR images of different types, yielded tumor necrosis estimations with not only a higher accuracy but also variance, while minimizing the maximum absolute error ensured minimum variance in sacrifice of accuracy. An alternative herein would have been to find a middle ground between the two optimization measures by combining them. For instance, an objective function such as *f* = *w*_avg_*f*_avg_ + *w*_max_*f*_max_ could be formed, where *w*_avg_ and *w*_max_ would be optimized (along with *w*_*k*_) in search of the minimum value of *f*. This would be a part of the authors’ continuing effort to further refine the current model as more imaging data becomes available. In future studies, more sophisticated image fusion and classification techniques such as neural networks shall be considered, as they become feasible with more assessable imaging data.

Due to issues such as incomplete mapping of histologic tumor necrosis, which results from unavoidable tissue shrinkage and loss during specimen processing for histopathology, and unattainability of a perfect histologic-MR image co-registration, it remains to be seen whether a feasible version of the current classification model could be obtained by performing the weight optimization based on a spatial mapping of tumor viability (e.g., through maximizing the Dice coefficient) instead of an overall percentage of tumor necrosis. In addition, almost all patients enrolled were good responders with higher than 90% tumor necrosis; this fact might possibly skew the optimization outcome towards identifying tumor necrosis. Furthermore, due to missing or lost data at random (i.e., as a result of technical or logistic glitches), the sample sizes were different in the models developed using conventional MRI versus advanced MRI. It was unfortunate as it led to a loss of statistical power in modeling, but it was believed that the analysis could be run with the remaining data without introducing bias, for the random loss of data could reasonably be expected not to skew the study’s results. Nevertheless, given the limited sizes of the samples, it is possible that a difference in performance could be driven by the change in both the sample size and the variation of the data itself. Only with more testing data one could verify the true validity of the current models. As mentioned earlier, the currently available imaging data is indeed limited (*n* = 10 patients), a direct consequence of slow progress due to limitations such as rarity of lesion, variability in histology and anatomy, and inconsistency of MRI sequences acquired. Notwithstanding, the results are promising and pointing to the prognostic value of utilizing MRI, both conventional and advanced, for evaluating tumor response in patients with osteosarcoma.
